# The After-Care of the Wounded

**Published:** 1915-04-17

**Authors:** Edwin L. Ash

**Affiliations:** 24 Harley Street, W.


*To the Editor of* The Hospital.

### The After-Care of the Wounded.

*To the Editor of* The Hospital.

Sir,—In recent issues (March 27 and April 10) The Hospital has drawn attention to the urgent necessity of making ample provision for invalided soldiers in respect of massage, mechano-therapeutics, and baths. Considering the numbers almost certain to be afflicted with the "rheumatism, fibrositis, and more or less allied conditions" referred to, it is to be hoped that the authorities will quickly see the advisability of acting in accordance with your suggestions. But I trust that those medical officers to whom is deputed the direction of these auxiliary methods of treatment will not overlook the benefits to be derived from the time-honoured methods of counter-irritation, which have lately fallen into disuse through misunderstanding of their scientific basis. In the various painful disorders of joints and muscles that very frequently follow wounds and exposure counter-irritation may be strongly relied on as a very valuable therapeutic adjunct to massage, baths, and mechano-therapeutics, always provided it be applied systematically, continuously, and with due regard to the anatomy of the neuro-muscular system. Haphazard use of blisters and stimulating liniments means a shot in the dark. Careful counter-irritation with definite intention to influence a particular nerve-track is a scientific measure. Moreover, not only may pain be relieved in this way, but the process aids in the absorption of those fibrous deposits which are so troublesome in chronic fibrositis, including in that term the rheumatism and neuritis one hears so much of in this damp climate. Space limitations preclude description of methods, but the blister, cautery, static electricity (breeze or sparks), and multiple acupuncture may be mentioned as particularly convenient and worthy to rank with measures, to use your own phrase, "tending to shorten convalescence, and to hasten the return of the worker to his position in the general scheme of things," such as we are all on the look-out for just now.—Faithfully yours,

Edwin L. Ash, M.D. Lond.

24 Harley Street, W., April 11, 1915.

